# Assessing Visual Crowding in Participants With Preperimetric Glaucoma Using Eye Movement and Manual Response Paradigms

**DOI:** 10.1167/tvst.13.9.8

**Published:** 2024-09-05

**Authors:** Dilce Tanriverdi, Khaldoon O. Al-Nosairy, Michael B. Hoffmann, Frans W. Cornelissen

**Affiliations:** 1 Laboratory for Experimental Ophthalmology, University of Groningen, University Medical Center Groningen, Groningen, Netherlands; 2 Section for Clinical and Experimental Sensory Physiology, Ophthalmic Department, Otto-von-Guericke University, Magdeburg, Germany; 3 Center for Behavioral Brain Sciences, Magdeburg, Germany

**Keywords:** preperimetric glaucoma, saccadic eye movements, peripheral crowding

## Abstract

**Purpose:**

Crowding is the inability to distinguish objects in the periphery in the presence of clutter. Previous studies showed that crowding is elevated in patients with glaucoma. This could serve as an indicator of the functional visual performance of patients with glaucoma but at present appears too time-consuming and attentionally demanding. We examined visual crowding in individuals with preperimetric glaucoma to compare the potential effectiveness of eye movement–based and manual response paradigms.

**Methods:**

We assessed crowding magnitude in 10 participants with preperimetric glaucoma and 10 age-matched controls. Crowding magnitudes were assessed using four different paradigms: a conventional two-alternative forced choice (2AFC) manual, a 2AFC and a six-alternative forced choice (6AFC) eye movement, and a serial search paradigm. All paradigms measured crowding magnitude by comparing participants’ orientation discrimination thresholds in isolated and flanked stimulus conditions. Moreover, assessment times and participant preferences were compared across paradigms.

**Results:**

Patients with preperimetric glaucoma exhibited elevated crowding, which was most evident in the manual-response paradigm. The serial search paradigm emerged as the fastest method for assessing thresholds, yet it could not effectively distinguish between glaucoma and control groups. The 6AFC paradigm proved challenging for both groups.

**Conclusions:**

We conclude that patients with preperimetric glaucoma demonstrate heightened binocular visual crowding. This is most effectively demonstrated via the 2AFC manual response paradigm. The additional attentional demand in eye movement paradigms rendered them less effective in the elderly population of the present study.

**Translational Relevance:**

Our findings underscore both the value and the complexity of efficiently evaluating crowding in elderly participants, including those with preperimetric glaucoma.

## Introduction

Glaucoma stands as a prominent cause of irreversible blindness on a global scale.^1^ It is characterized by the gradual degeneration of retinal ganglion cells and optic nerve fibers. Preperimetric glaucoma refers to the early stage of the disease where optic nerve damage is present but visual field loss, as detected by standard perimetry, has not yet occurred.^2^^,^^3^ Despite lacking apparent visual field loss, patients with preperimetric glaucoma experience a lower vision-related quality of life.^4^

Visual crowding, as described by Bouma,^5^ refers to the reduced ability to recognize objects when they are surrounded by visual clutter. This phenomenon represents a fundamental limitation in human vision, impacting tasks such as object recognition, reading, driving, and visual search.^6^ These crowding effects are reported to be stronger in people with certain visual deficits such as amblyopia,^7^ nystagmus,^8^ and, more recently, glaucoma.^9^^–^^13^ Investigating crowding within the context of these developmental and visual impairments is crucial for understanding these deficits and devising effective treatments, as emphasized by de Vries et al.^14^ Furthermore, these studies provide insights into the underlying mechanisms of crowding.

Research has indicated that individuals with glaucoma experience heightened levels of crowding in both their parafoveal^10^^,^^15^ and peripheral vision.^9^ Ogata et al.^9^ in their study showed that when measured monocularly, patients with glaucoma with mild to moderate visual field loss showed higher peripheral crowding compared to age-matched controls even when the stimulus was presented only in the intact areas of their visual field. Moreover, they found a significant correlation between the amount of nerve tissue loss as measured by optical coherence tomography and the magnitude of crowding. They suggested that the decrease in retinal ganglion cells in glaucoma results in reduced sampling, consequently leading to expanded areas of summation.^16^ Shamsi et al.^10^ showed that participants with glaucoma showed parafoveal elevated crowding in their worse eye compared to their better eye. Moreover, when measured binocularly, patients with glaucoma had higher crowding than age-matched controls. The linkage between reduced ganglion cell density, indicated by a thinning of the retinal nerve fiber layer (RNFL) and ganglion cell layer (GCL), and an increased crowding zone has been further demonstrated in previous literature.^11^^,^^17^

Considering the evident RNFL thinning in patients with preperimetric glaucoma prior to vision loss,^18^ one might expect elevated crowding in these patients. If so, crowding could serve as a valuable tool in assessing the functional visual performance of patients with preperimetric glaucoma. It may help to understand the challenges these patients encounter in daily visual tasks, such as driving and recognizing surrounding objects.^6^ Elevated crowding has been shown to be closely related to decreased reading speed, a smaller functional field of view, and difficulty recognizing faces in patients with glaucoma.^12^^,^^13^^,^^19^ However, current methods for studying crowding primarily rely on conventional psychophysical techniques that, while reliable, are also time-consuming and attention demanding. These methods often require participants to press buttons, follow complex instructions, and maintain fixation.^20^^–^^22^ Such approaches therefore pose significant challenges for clinicians, researchers, and patients alike when attempting to assess crowding.

In light of these challenges with conventional methods, there is an opportunity to explore the potential of eye movements as an additional tool that could enhance the assessment of crowding in patients with glaucoma. Previous research has used eye movements to assess various aspects of functional vision more quickly and intuitively.^23^^,^^24^ Additionally, eye movements have been employed as a “built-in response method” to replace manual responses when measuring crowding.^25^^–^^27^ Manual responses are defined as responses recorded using an external device such as keyboard or mouse inputs in this context. Following this line of research, we previously developed a collection of crowding paradigms that utilize eye movements as a response measure. We showed that within a population of healthy young participants, paradigms utilizing eye movements as a response measure accurately measure crowding and do so more efficiently than conventional paradigms that rely on manual response.^28^

We aimed to investigate whether binocular crowding is elevated, specifically in patients with preperimetric glaucoma compared to age-matched controls. This is still an open question, as previous studies focused on comparing monocular peripheral crowding between individuals with either moderate or intermediate stages of glaucoma to healthy controls^9^^,^^10^ or on comparing binocular parafoveal crowding in individuals with either moderate or intermediate stages of glaucoma versus age-matched controls.^10^ Moreover, we aimed to investigate whether eye movement–based paradigms may be more effective compared to a conventional manual response paradigm in assessing crowding in patients with preperimetric glaucoma. For this purpose, we compared four paradigms (two-alternative forced choice [2AFC] manual, 2AFC eye movement, six-alternative forced choice [6AFC] eye movement, and a serial search paradigm) in terms of the observed crowding magnitude, measured by orientation discrimination thresholds, assessment time, and participant preferences. We used reestimated crowding magnitude, orientation discrimination thresholds, and assessment time measurement, calculated by means of an efficiency analysis. This method mirrors our prior study,^28^ where we reestimated these values by establishing a confidence interval cutoff. This allows us to identify the most efficient paradigm in terms of the number of trials and the time needed to achieve reliable results.

## Methods

### Participants

Utilizing an a priori power analysis derived from the effect size outlined in the study by Shamsi et al.,^10^ the study enrolled a total of 20 participants. Among these, 10 individuals were allocated to the preperimetric glaucoma group (seven females; mean age = 71.3, SD = 7.13), while the remaining 10 were assigned to the age-matched control group (five females; mean age = 69.1, SD = 5.22). All participants gave their written informed consent to participate in the study. Our study followed the tenets of the Declaration of Helsinki and was approved by the medical ethical assessment committee of the Otto-von-Guericke University Hospital.

#### Ophthalmological Characterization

All participants underwent standard ophthalmologic exams, including refractive corrections, stereo-tests, intraocular pressure measurements, and standard visual field (VF) tests. All healthy controls and patients with glaucoma had best-corrected visual acuity <0.2 (logarithm of minimal angle of resolution), normal Lang stereo-test, and no other diseases affecting vision.

Patients with preperimetric open-angle glaucoma met the following criteria after the American Academy of Ophthalmology guidelines for primary open-angle glaucoma^29^: (1) interocular pressure >21 mm Hg, (2) definite optic disc or RNFL abnormalities consistent with glaucoma, and (3) no VF defects in standard automated perimetry.

### Paradigms and Stimuli

#### Stimuli and Equipment

Visual fields were evaluated using the 24-2 standard algorithm of the Humphrey Field Analyzer (HFA) 3 (Carl Zeiss Meditec AG, Jena, Germany). The test stimulus, measuring 4 mm² in size (equivalent to a size III Goldmann stimulus, i.e., 0.43°), was presented for a duration of 0.2 seconds.

Crowding stimuli were presented on a light-emitting diode backlight monitor (Samsung2494 [Samsung Digital City, Suwon, South Korea] with a refresh rate of 60 Hz and pixel resolution of 1920 × 1080). The measured mean luminance of the screen was 61 cd/m². MATLAB software version 2020a (MathWorks, Natick, MA, USA) was used with the Windows 10 Enterprise operating system to execute the experiments. The visual stimuli were programmed using the PsychToolbox extension.^30^^–^^32^ Eye position signals were recorded with a LiveTrack Lightning eye tracker (Cambridge Research Systems, Cambridge, UK) at a sampling rate of 500 Hz. MATLAB functions provided by the manufacturer were used to integrate the eye tracker functionality into the experimental script and to calibrate the eye tracker using the built-in 9-point calibration procedure. The display was viewed binocularly from a distance of 61 cm. A fixed head position was ensured by using a head and chin rest.

**Figure 1.**
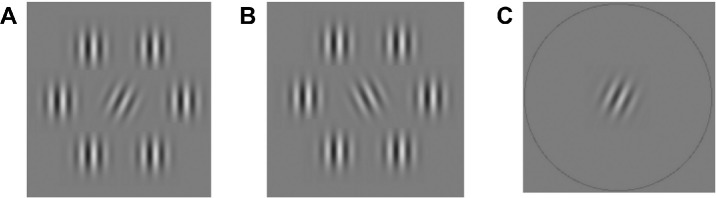
Stimulus arrays used in different conditions. (**A**) An example stimulus array with the target surrounded by flankers with the same spatial frequency as the target. (**B**) A nontarget surrounded by flankers with the same spatial frequency. (**C**) A target in the isolated condition.

Within each paradigm, participants were required to indicate the position of a target among one or more nontargets. The stimuli comprised Gabor patches (1° diameter, 5 cycles per degree spatial frequency), presented either in isolation or encircled by six Gabor patches serving as flankers (75% Michelson contrast, 90° tilt, Figs. 1A, 1B). In cases of isolation, a circle surrounded the central element (50% Michelson contrast, Fig. 1C). To maximize crowding effects in all paradigms, we set the contrast of the target and nontarget Gabors lower than that of the flankers.^33^^,^^34^ The specific spatial frequency was selected to keep the contrast sensitivity at peak performance ranges.^35^^,^^36^ The tilt angle of the central Gabor was manipulated (90° ± [0–45]°), with a clockwise tilt designating it as the target and an anticlockwise tilt as the nontarget. The number and screen positions of stimulus arrays varied across paradigms. Across all paradigms, the eccentricity of the stimulus array remained at 6° to fixation, and the center-to-center distance between the target and flankers was fixed at 1°.

**Figure 2.**
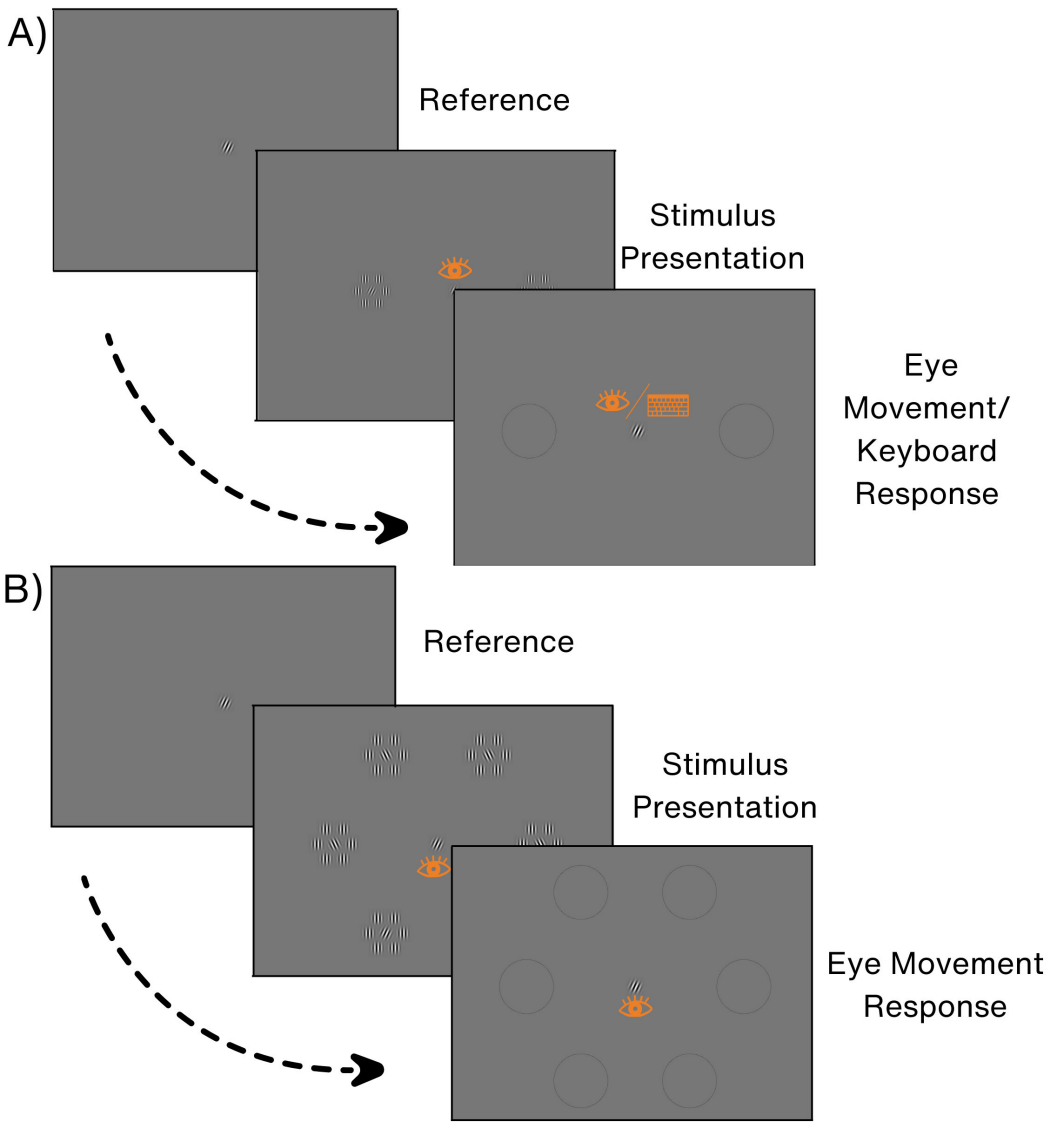
The procedure of the 2AFC paradigm in **A** and the 6AFC paradigm in **B**. The eye icon represents fixation during the stimulus presentation panels and saccadic response in the response panels. The keyboard represents a button press as a manual response. Participants either made a saccade toward the location of the stimulus array with the right-tilted central Gabor patch or pressed the arrow button indicating the location.

**2AFC and 6AFC Paradigms:** In the 2AFC paradigm, two stimulus arrays were positioned along the horizontal meridian. The distance of the central element of the array to the fixation was 6° of eccentricity (see Fig. 2A). The target was randomly positioned to the left or right of fixation. A reference Gabor was consistently presented in the middle of the screen throughout the experiment, serving as a fixation point. Participants were explicitly instructed to maintain fixation on this central Gabor. The orientation of this central Gabot patch always matched the target.

After making sure the participants’ gaze was on the fixation spot, the stimuli were presented for a duration of 700 ms. Participants were asked to fixate in the central Gabor during the stimulus presentation. However, if the participants broke fixation, the target and nontarget were deleted from the screen after 150 ms. The participant's responses in such trials were considered valid. This approach was utilized to mitigate data loss attributed to fixation breaks and simultaneously minimized the impact of eye movements occurring during the presentation of stimuli. After stimulus presentation, two black circles appeared on the screen at the previous locations of the stimulus arrays. In the manual response paradigm, participants indicated their response by pressing either the left or the right arrow key on the keyboard. In the eye movement response paradigm, they responded by moving their eyes to the location of one of the black circles on the screen. Participant's response was registered when an eye movement was made to a stimulus array and the eye position entered the predetermined area of interest (AOI). There was no time restriction for participants to respond after the stimulus disappeared from the screen. The 6AFC paradigm was similar to the 2AFC paradigm. However, in this variant, six rather than two stimulus arrays were arranged in a hexagonal shape, each positioned at an eccentricity of 6° to the central element. In this paradigm, participants only responded via an eye movement toward the stimulus (see Fig. 2B). In all paradigms, the orientation discrimination threshold was determined utilizing QUEST.^37^ This involved evaluating participant accuracy at 75% correct performance, with an assumed guess rate of 0.17 in the 6AFC paradigm and 0.5 in the 2AFC paradigm. QUEST had a range expanding from 0° to 50° for its probability density function (PDF). However, we limited the paradigm not to show orientations larger than 45° or smaller than 0° during the experiment. Therefore, if a participant could not perform a certain paradigm, they would be presented with a 45° tilt orientation for the target and nontarget during the experiment. However, the highest score they could attain from the QUEST would be 50°. This would happen because QUEST would put low probabilities on all the presented and wrongly answered orientations in its PDF.

#### Serial Search Paradigm

**Figure 3.**
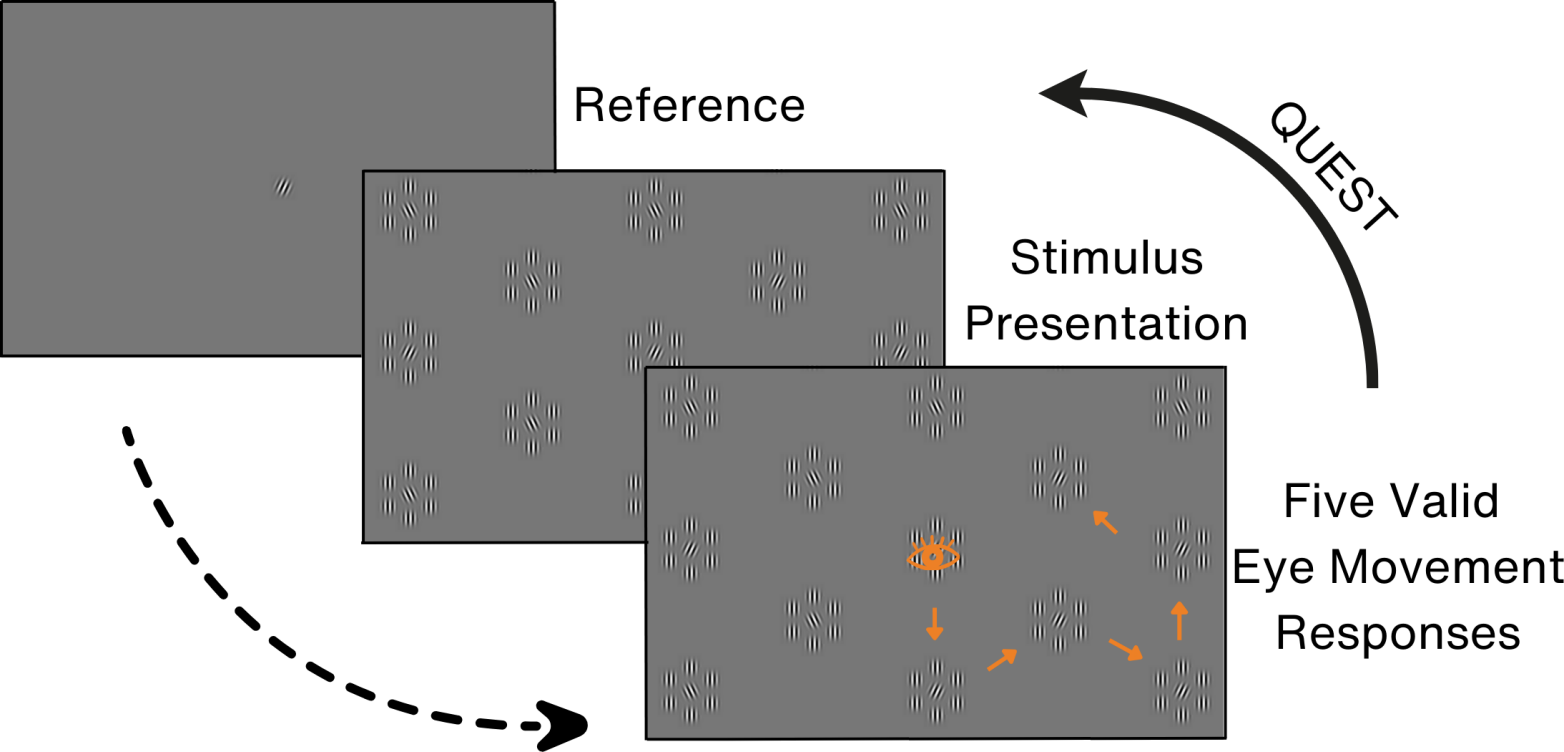
Procedure of the serial search paradigm. The eye icon represents a saccadic response. The *orange arrows* represent a hypothetical path a participant can take during the presentation of one target orientation. The orientation of all central Gabors shown on the display screen is updated based on a participant’s recognition performance. Note that the stimulus presentation shows only a part of the screen to increase the visibility of the image and not all of the stimulus arrays.

In this paradigm, a grid comprising 28 stimulus arrays was displayed on the screen, ensuring uniform center-to-center distances between the arrays, corresponding to a 6° eccentricity of the central elements of arrays. Of the arrays on the screen, half contained a target while the other half contained a nontarget (see Fig. 3). The selection of arrays with targets followed a semirandom pattern, guaranteeing that half of the arrays in any given column contained a target. Notably, the central array of the screen always featured a target. This served as a starting point for each screen refresh.

The experimental procedure started with the presentation of a reference Gabor at the screen’s center. This Gabor patch served as an initial fixation spot, and its orientation matched the target elements. After making sure participants’ gaze was on the reference Gabor, as indicated by the eye-tracking signals, the stimulus screen was presented. Participants were instructed to continuously shift their gaze only to arrays where they perceived the presence of a target. Similar to the forced-choice eye movement paradigms, a valid response was counted when an eye movement was made to a stimulus array and the eye position entered the predetermined AOI (see the Eye Movement Analysis section for further detail) for that stimulus array.

Crucially, once a stimulus array had been fixated, it was excluded from the “valid response” tool, yet remained visible on the screen. Consequently, revisiting a previously chosen array was possible but did not count as a valid response. This was to prevent repetitive gazing at the same arrays from affecting performance. This strategy aimed to minimize potential fluctuations in the guess rate that could occur if participants were able to recall visited arrays. However, deleting locations from the response pool after multiple responses would also affect the overall guess rate. Therefore, to discourage repetitive viewing and minimize fluctuations in guess rates caused by removing visited locations from the response pool, the stimulus screen was updated after every five valid responses. For such an update of the screen, the stimulus screen was replaced by a new one in which the locations of the target and nontarget Gabors were reassigned, following the same procedure as described above. Additionally, the orientations of the central Gabor patches were updated based on the value indicated by QUEST. Notably, in this paradigm, QUEST receives five consecutive responses for the same orientation angle before being prompted for a new orientation angle. These calculations were based on participant responses to the previously presented orientation angle.

The participant’s orientation discrimination threshold in each condition was established at 75% correct performance. The guess rate was fixed at 50%, representing the average guess rate across the entire stimulus screen. While the number of possible arrays to choose from varied across different screen locations, we opted for a consistent guess rate to avoid having to adjust it based on the participant’s gaze location. For instance, at the screen center, participants could choose from six distinct arrays, while the number of choices decreased toward the edges and the corners of the screen.

### Procedure

The study was conducted at the Ophthalmic Department of the Otto-von-Guericke University Hospital. As an initial step, each participant underwent a comprehensive visual screening test, which included assessments of visual acuity, standard automated perimetry, and stereopsis. Each participant had proper refractive correction for the testing distance, determined by the optometrist who was present during the experiment. After the screening, participants were brought to a dimly lit room, where they completed the four crowding paradigms, including two 2AFC paradigms with either manual or eye movement responses, the 6AFC paradigms with eye movement responses, and the serial search paradigm in four separate blocks in random order. Within a block, two conditions—isolated and flanked—were interleaved and presented in a random order, culminating in a total of 200 trials per block.

At the beginning of each block, participants were briefed on the specific paradigm they were about to engage in, and a training session followed. Prior to each block, the participants underwent a calibration procedure for the eye tracker. Every 100 trials, participants were granted a chance to rest their eyes while keeping their heads on the headrest, while a 10-minute break was available after completing two blocks.

Upon concluding the experiment, participants were tasked with completing a questionnaire tailored to each paradigm. This survey comprised five questions, each answered on a 1-to-5 Likert scale and a rank question. The initial question evaluated the perceived difficulty of the paradigm, while the second one assessed the participants’ experienced level of fatigue upon completion. The third question assessed the perceived demands imposed by the paradigm. The fourth question inquired about the perceived level of attention required throughout the paradigm. The fifth question focused on the amount of effort participants felt was necessary for successfully performing and completing the paradigm. Participants were also asked to rank the paradigms from most to least preferred, with 1 being the most preferred and 4 being the least preferred. Lower scores from the questions listed above meant higher preference for the paradigms in the study. As a final question, the participants were asked whether they understood the instructions of each paradigm they had completed. The score from this question was not added to the total score and was used as a quality check for clarity of instructions for each paradigm. See Supplementary Table S1 for the full questionnaire. While answering each question, participants were shown a representative picture of the paradigms they had completed to remind them of the paradigms in the study. Participants were aware of the overall aim of the study but were not aware of the specific expectations related to each paradigm while answering these questions. They were allowed to pose questions to the experimenter for clarification while responding to the questionnaire items.

### Eye Movement Analysis

For the analysis of eye movements, we employed the 9-point calibration procedure provided by Cambridge Research Systems’ LiveTrack Lightning MATLAB toolbox before the start of each block. Real-time recording and analysis of eye movements were conducted throughout the experiment. Both eyes were recorded, and the average of both eyes’ x and y coordinates was used to locate the gaze location during the experiment. In paradigms involving eye movement responses, a rectangular AOI measuring 1.5° in both width and height was designated at the location of each central Gabor patch. Whenever a participant’s gaze intersected with this AOI, the system recorded a response. In all paradigms, the stimulus was shown to the participants after verifying that the participant's gaze landed within the reference AOI at the location of the Gabor patch in the middle of the screen. Note that this verification happened after each response in the forced-choice paradigms and after five valid responses in the serial search paradigm.

### Statistical Analysis and Reestimating Thresholds and Paradigm Duration

In our study, three primary dependent variables were considered. The initial variable, crowding magnitude, was defined as the orientation discrimination threshold in the flanked condition divided by the isolated condition. The second dependent variable, reestimated assessment time, represented the time taken to complete each paradigm, with breaks after the 100th trial subtracted from the total. Finally, participants’ preference for paradigms, assessed via total questionnaire scores, constituted our third dependent measure. Higher points meant less preference toward a paradigm.

Efficiency is a crucial aspect of our paradigm comparison, and we acknowledge that it can be influenced by factors such as the number of alternatives (targets versus nontargets) in each paradigm. To assess the efficiency of each paradigm, we used a similar method to our previous study,^28^ where we established an a posteriori cutoff confidence interval (CI) by examining the mean CI plots across all paradigms. We chose the CI at which stability was observed across all conditions and paradigms except the 6AFC paradigm as our cutoff point. The 6AFC paradigm was not included in this decision because participants were not able to perform the paradigm (see the Results section). For each participant and paradigm, we calculated the number of trials needed to reach this CI for their threshold estimations. Using these figures, we reestimated the thresholds, considering only the trials necessary to reach the CI cutoff. This process entailed supplying QUEST with responses until the required number of trials was completed. Additionally, we recalculated the duration for each paradigm using the following formula:

$$\begin{array}{ccc} & & \;Recalculated\;Duration=Original\;Duration\;\\ & & \;\times \frac{Total\;Number\;of\;Trials\;to\;Reach\;Reliable\;Threshold}{Total\;Number\;of\;Trials}. \end{array}$$

In the main text, we will present the results using these reestimated values for orientation thresholds, crowding magnitude, and assessment time values. The comparison of the original values to the reestimated values as well as the graphs for the original values can be found in the supplementary material.

To evaluate each paradigm and participant group, an initial 3 × 2 × 2 mixed analysis of variance (ANOVA) was conducted with paradigm type (2AFC eye movement, 2AFC manual, serial search) and crowding condition (flanked and isolated) as within-subject variables and participant group (control and glaucoma) as a between-subject variable on orientation discrimination thresholds. This allowed us to examine the simple main effect of crowding conditions on each paradigm for each participant group, elucidating whether the presence of flankers led to higher orientation thresholds across paradigms and participant groups. Before conducting an ANOVA on crowding magnitude, the crowding magnitude values were log-transformed to achieve a normal distribution. Subsequently, another mixed ANOVA was carried out on log-transformed crowding magnitude values with paradigm type (2AFC eye movement, 2AFC manual, serial search) as a within-subject variable and participant group (control versus glaucoma) as a between-subject variable.

The differences between participants with preperimetic glaucoma and controls in terms of age, visual acuity, mean visual field deviations (MDs), and visual field indices (VFIs) were analyzed using the Mann–Whitney *U* test. A correlation analysis was conducted between the crowding magnitude and localized sensitivities from the perimetry analysis, as well as RNFL thickness values. Please see the Supplementary Figure S3 for the details of these analyses.

To analyze assessment time, a mixed ANOVA was conducted, incorporating paradigm type (2AFC eye movement, 2AFC manual, and serial search) as a within-subject variable and participant group (control versus glaucoma) as a between-subject variable. Notably, the 6AFC paradigm with eye movement responses was excluded from all of the above analyses due to most participants reaching floor-level performance in both crowded and isolated conditions, as further detailed in the Results section. To investigate whether there was a difference among participants who exhibited a floor effect in the rest of the paradigms, we conducted a χ^2^ test on the number of participants with scores exceeding 45° (highest tilt orientation shown) in the original orientation discrimination thresholds. Counts from both the isolated and flanked conditions for each task, as well as from the control and glaucoma groups, were combined for this analysis.

To analyze the questionnaire data, we employed a mixed-method approach incorporating both Rasch analysis and a mixed ANOVA to explore differences in responses across conditions and groups. First, we conducted a Rasch analysis using the method of successive dichotomizations from the “msd” R package^38^ to transform the Likert scale responses into person measures. Person measures, expressed in logits, represent a continuous measure of the latent trait assessed by the questionnaire. In the context of our study, higher person measures indicate a lower preference for the paradigm, while lower person measures indicate a higher preference for the paradigm. For three participants (two controls and one with glaucoma), we could not calculate the person measures due to insufficient variability in their responses. As a result, these participants were excluded from the subsequent ANOVA. We fitted a mixed-effects model (2 × 4) using the lme4 package in R, accounting for repeated measures within subjects by including random intercepts for each participant. For further investigation with post hoc tests, we performed pairwise comparisons using the “emmeans” package, adjusting for multiple comparisons with the Bonferroni correction.

## Results

Our results revealed no difference between participants with preperimetric glaucoma and controls in terms of age and visual acuity. There was a difference between these groups in terms of MDs and VFIs obtained from the 24-2 HFA (see Table 1).

**Table 1.** Overview of Participant Characteristics

Characteristic	Control (Median)	Glaucoma (Median)	*P* Value	Effect Size
Age	67.5	71.5	0.26	−0.31
BCVA OD (logMAR)	−0.1	0	0.22	−0.31
BCVA OS (logMAR)	−0.1	0	0.20	−0.32
MD OD (dB)	0.97	−1.31	0.009	0.68
MD OS (dB)	1.18	−0.85	0.002	0.84
VFI OD	1	0.98	0.007	0.68
VFI OS	0.99	0.98	0.002	0.76

BCVA, best-corrected visual acuity; dB, decibels; logMAR, logarithm of minimal angle of resolution; MD, mean visual field deviation of 24-2 SITA standard visual field; OD, right eye; OS, left eye; VFI, visual field index (0%–100%).

We initiated our analysis by examining the original orientation discrimination thresholds obtained from each QUEST run for every participant. As stated in the Methods, 45° was the highest tilt orientation shown to the participants in all paradigms. However, QUEST had a range expanding to 50° for mean threshold estimations. Consequently, if a participant could not perform a given paradigm, the highest score they could attain from the QUEST was 50. As shown in Table 2, almost all participants had threshold values above 45° in the 6AFC eye movement paradigm. Therefore, we opted to omit this paradigm from our further analyses concerning threshold and duration values.

**Table 2.** Number of Participants out of 10 Controls and 10 Patients with Glaucoma Who Reached Threshold Values Above 45° in All Paradigms

	Control, *n*		Glaucoma, *n*	
Paradigms	Flanked	Isolated	Flanked	Isolated
2AFC eye movement	3	1	4	0
2AFC manual	1	0	0	0
6AFC eye movement	9	8	10	10
Serial search	7	2	7	3

As indicated in the Methods and the Introduction, we will present the rest of the results using the reestimated values for orientation thresholds, crowding magnitude, and duration. We did not find a significant difference between the original and recalculated values for threshold and crowding magnitude variables overall (see Supplementary Table S2 and Supplementary Fig. S2).

**Figure 4.**
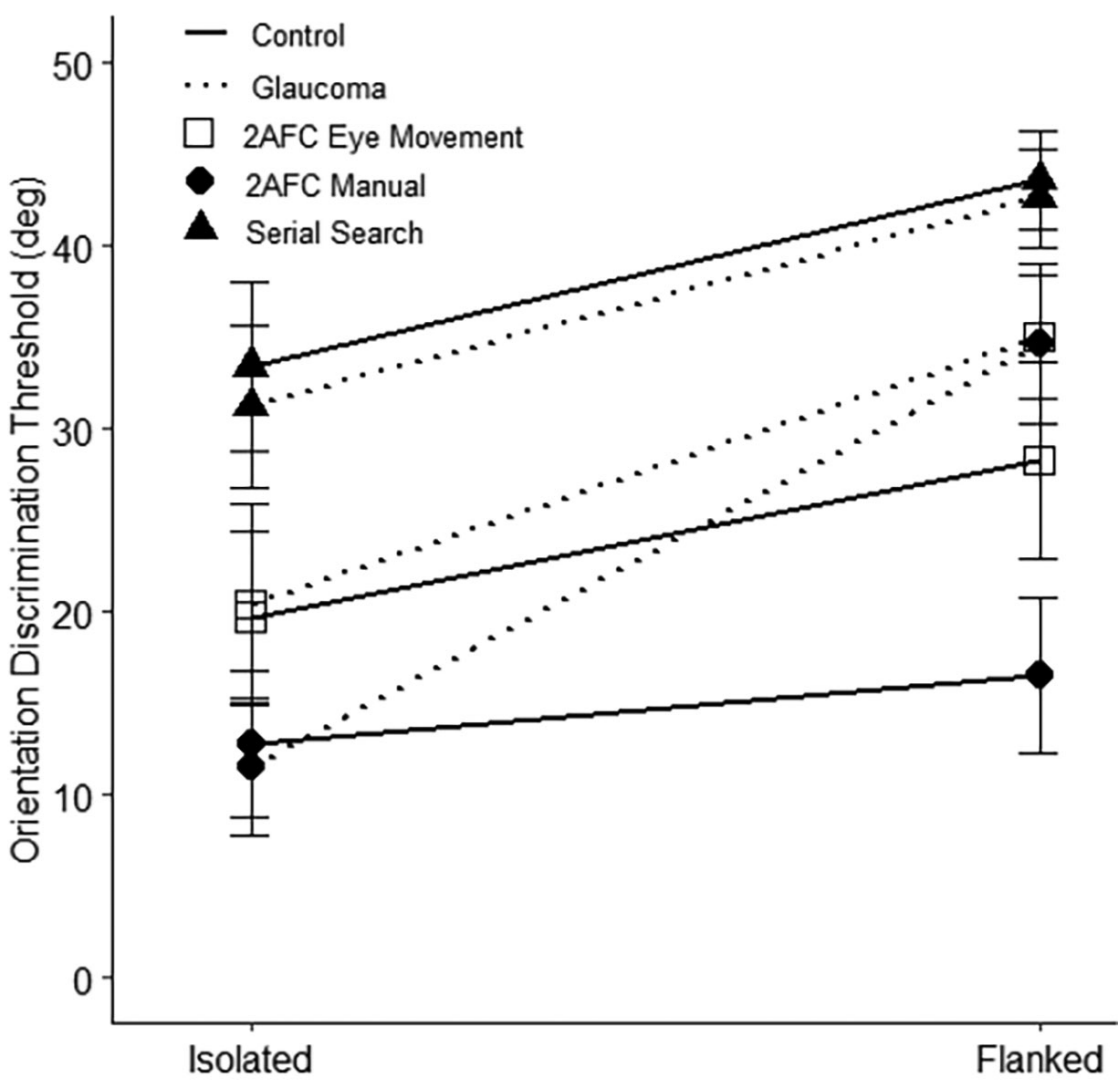
Mean orientation discrimination thresholds as a function of paradigm and participant group. The *shapes* are the mean while the *error bars* represent the standard error of the mean. In the glaucoma group, thresholds showed a difference between crowded and isolated conditions across all three paradigms. Meanwhile, in the control group, thresholds differed between crowded and isolated conditions in the 2AFC eye movement and serial search paradigms but not in the 2AFC manual paradigm.

We then aimed at determining the hallmark of visual crowding (i.e., whether the presence of flankers led to higher orientation thresholds across paradigms and participant groups). Figure 4 indicates that for all groups, the mean thresholds in the flanked conditions were higher than the isolated ones. However, the statistical significance of this result depended on both group and paradigm. A simple main effects analysis revealed that in the glaucoma group, there was a difference between the thresholds in the crowded and isolated conditions in all three paradigms (*F*(1, 18) = 12.46, *P* = 0.01 for the 2AFC eye movement paradigm; *F*(1, 18) = 23.21, *P* < 0.001 for the 2AFC manual paradigm; and *F*(1, 18) = 5.61, *P* = 0.04 for the serial search paradigm). In the control group, the thresholds in the crowded and isolated conditions differed from each other in 2AFC eye movement (*F*(1, 18) = 5.84, *P* = 0.04) and serial search paradigms (*F*(1, 18) = 5.77, *P* = 0.04) but not in the 2AFC manual paradigm ( *F*(1, 18) = 3.46, *P* = 0.10).

Our second analysis examined the presence of differences in crowding magnitude between paradigm types and participant groups. The results revealed a significant main effect of both group (*F*(1, 18) = 5.93, *P* = 0.03, η_2_^p^ = 0.25) and paradigm type (*F*(2, 36) = 4.09, *P* = 0.03, η_2_^p^ = 0.19). There was also a significant interaction between paradigm type and group (*F*(2, 36) = 4.94, *P* = 0.01, η_2_^p^ = 0.22). This indicates that our estimates of crowding magnitude differed between groups while the size of the estimated difference depended on the paradigm used. The post hoc analysis revealed that the crowding magnitude in the serial search paradigm was different from the manual response 2AFC paradigm (*t* = 2.80, *P* = 0.03, *d* = 0.69).

**Figure 5.**
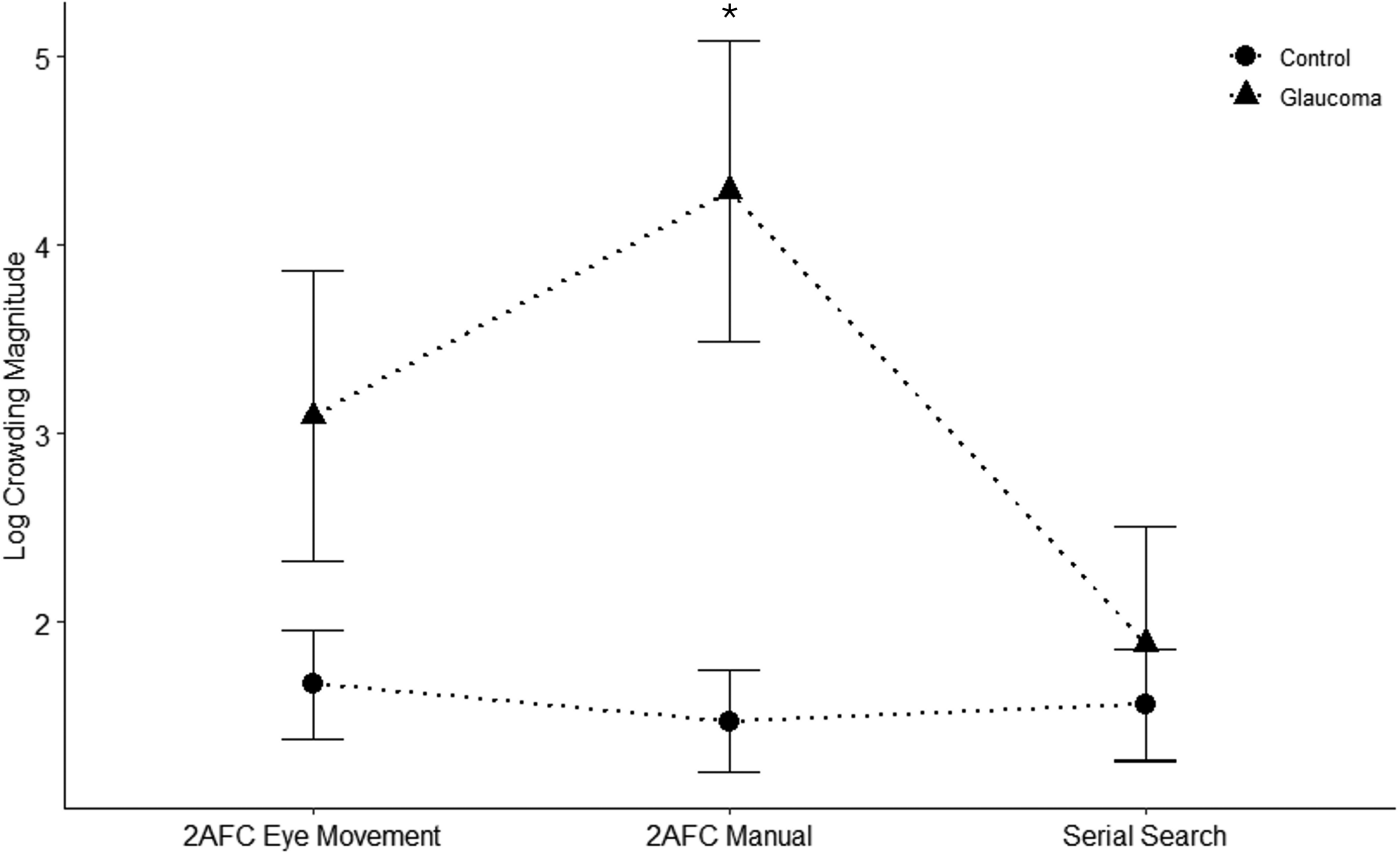
Crowding magnitude for 2AFC eye movement, 2AFC manual, and serial search paradigms. The *symbols* represent the mean while the *error bars* represent the standard error of the mean. The crowding magnitude for glaucoma and control groups significantly differed from each other in the 2AFC manual paradigm but not in 2AFC eye movement and serial search paradigms.

As shown in Figure 5, the custom contrasts based on our planned hypothesis testing revealed that the crowding magnitude for glaucoma and control groups significantly differed from each other in the 2AFC manual paradigm (*t*(41.13) = −3.69, *P* < 0.001). However, there was no significant difference in the crowding magnitude of glaucoma and control groups in neither the 2AFC eye movement (*t*(41.13) = −1.71, *P* = 0.1) nor the serial search paradigm (*t*(41.13) = −0.25, *P* = 0.81).

The results of the correlation between the 2AFC manual crowding magnitude, localized sensitivities, and RNFL thickness can be found in the supplementary material.

**Figure 6.**
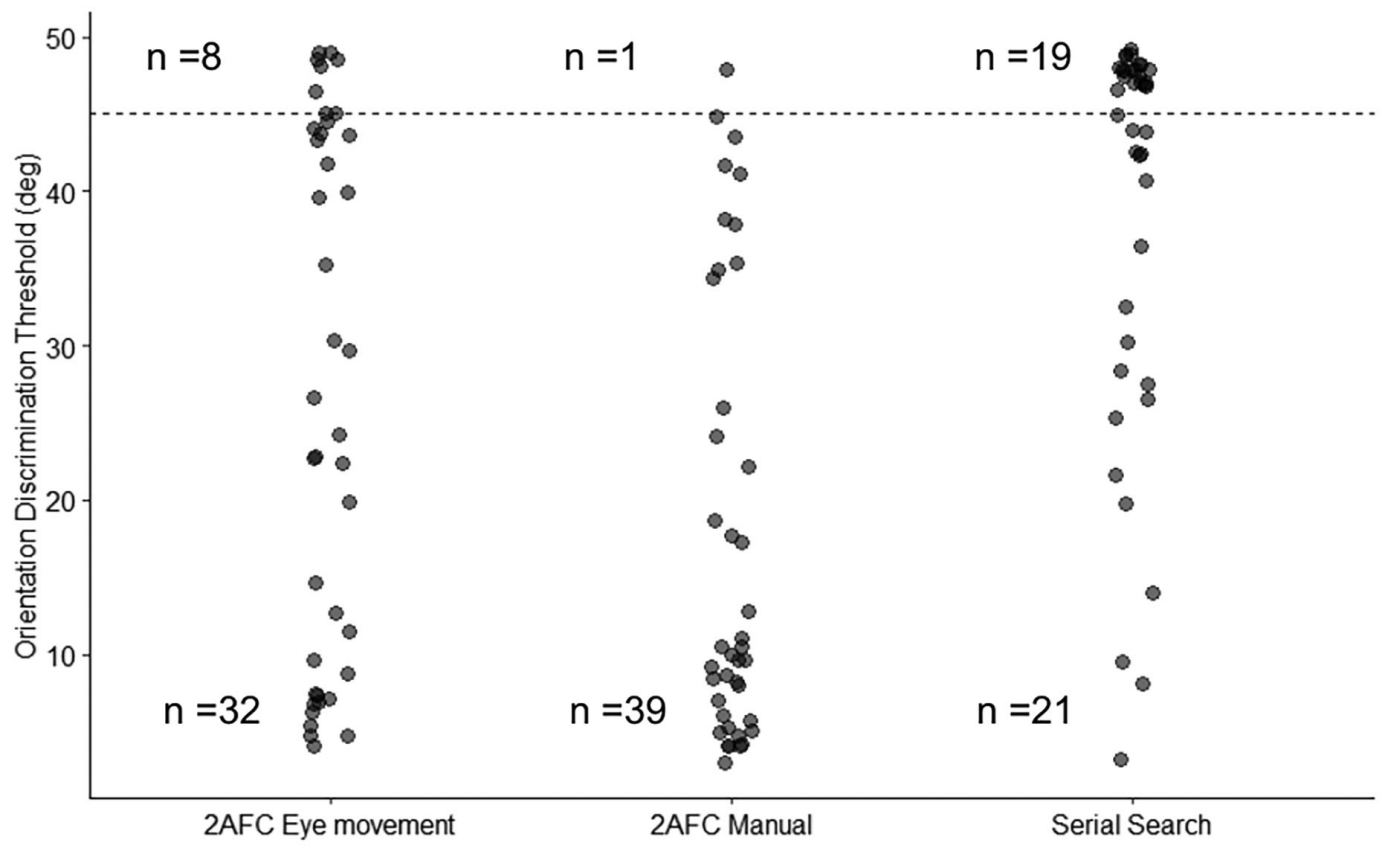
Participants’ orientation discrimination thresholds, above and below the floor level (indicated by the *striped line*) for all paradigms. The groups (controls versus glaucoma) and flanker conditions (flanked versus isolated) are combined.

We suspected that our inability to distinguish between the control and glaucoma groups in eye movement paradigms somehow related to the general increase in orientation discrimination thresholds observed in the eye movement paradigms, as illustrated in Figure 4, and an associated number of participants that reached the floor threshold, as illustrated in Table 2 and Figure 6. If a larger number of participants reached the floor level in these paradigms, it might suggest that the failure to find a difference in crowding magnitude might be due to the cognitive demands of the paradigm. It might have been too challenging for the groups investigated. Indeed, there was a difference between paradigms in terms of the number of participants that reached the floor-level threshold (χ^2^(2) = 17.64, *P* < .001).

**Figure 7.**
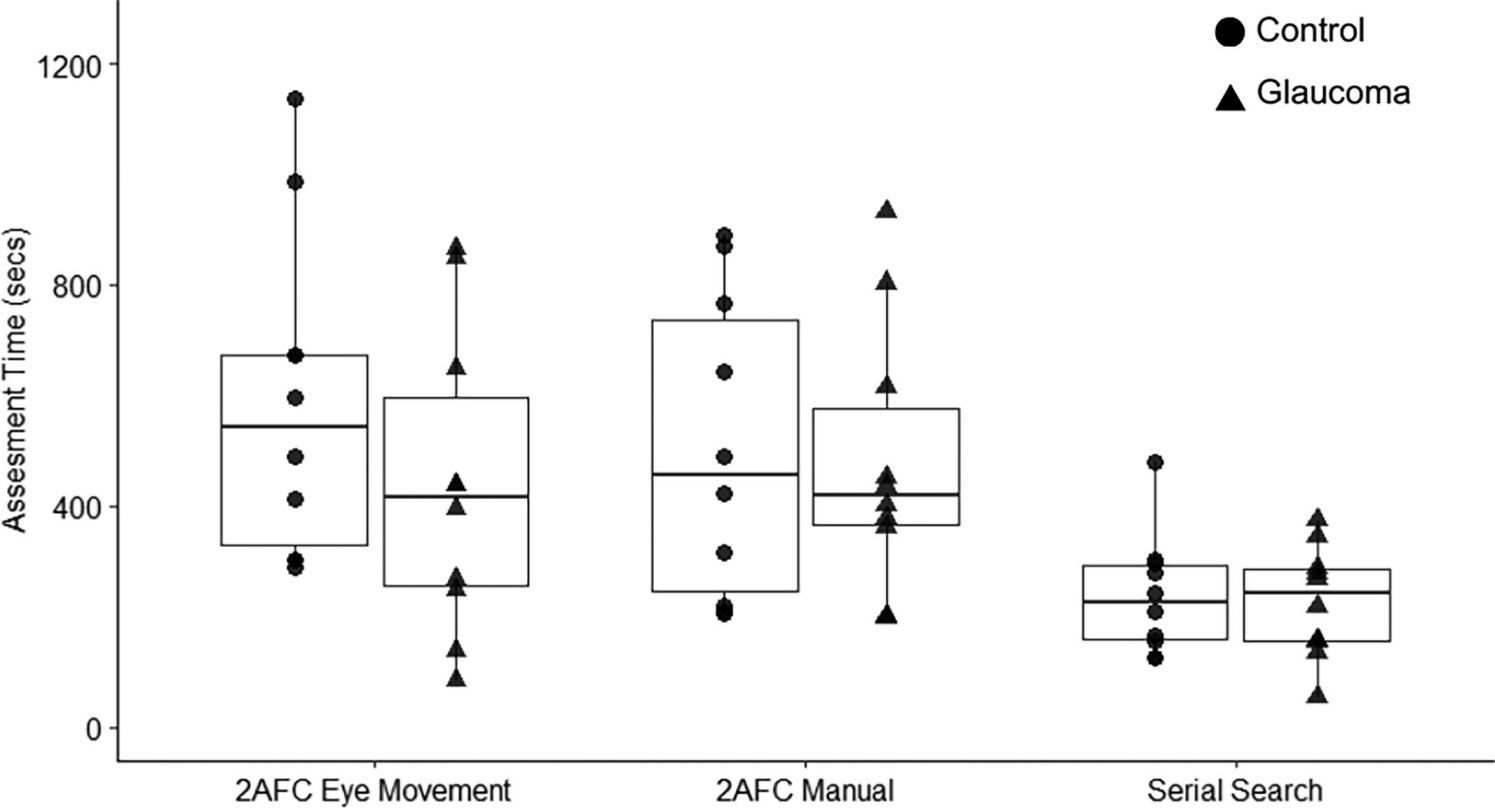
Assessment time in the 2AFC eye movement, 2AFC manual, and serial search paradigms. The serial search paradigm differs significantly from both the 2AFC manual and the 2AFC eye movement paradigms for both glaucoma and control groups.

After analyzing the orientation thresholds and crowding magnitude, we focused our analyses on assessment time. As can be seen in Figure 7, the serial search paradigm was the fastest out of the three paradigms at assessing thresholds. The results for assessment time revealed a significant difference between the serial search paradigm, on the one hand, and both the 2AFC manual (*t* = 3.80, *P* < 0.001, *d* = 1.14) and the 2AFC eye movement (*t* = 4.11, *P* = 0.001, *d* = 1.23) paradigms, on the other hand, respectively.

**Figure 8.**
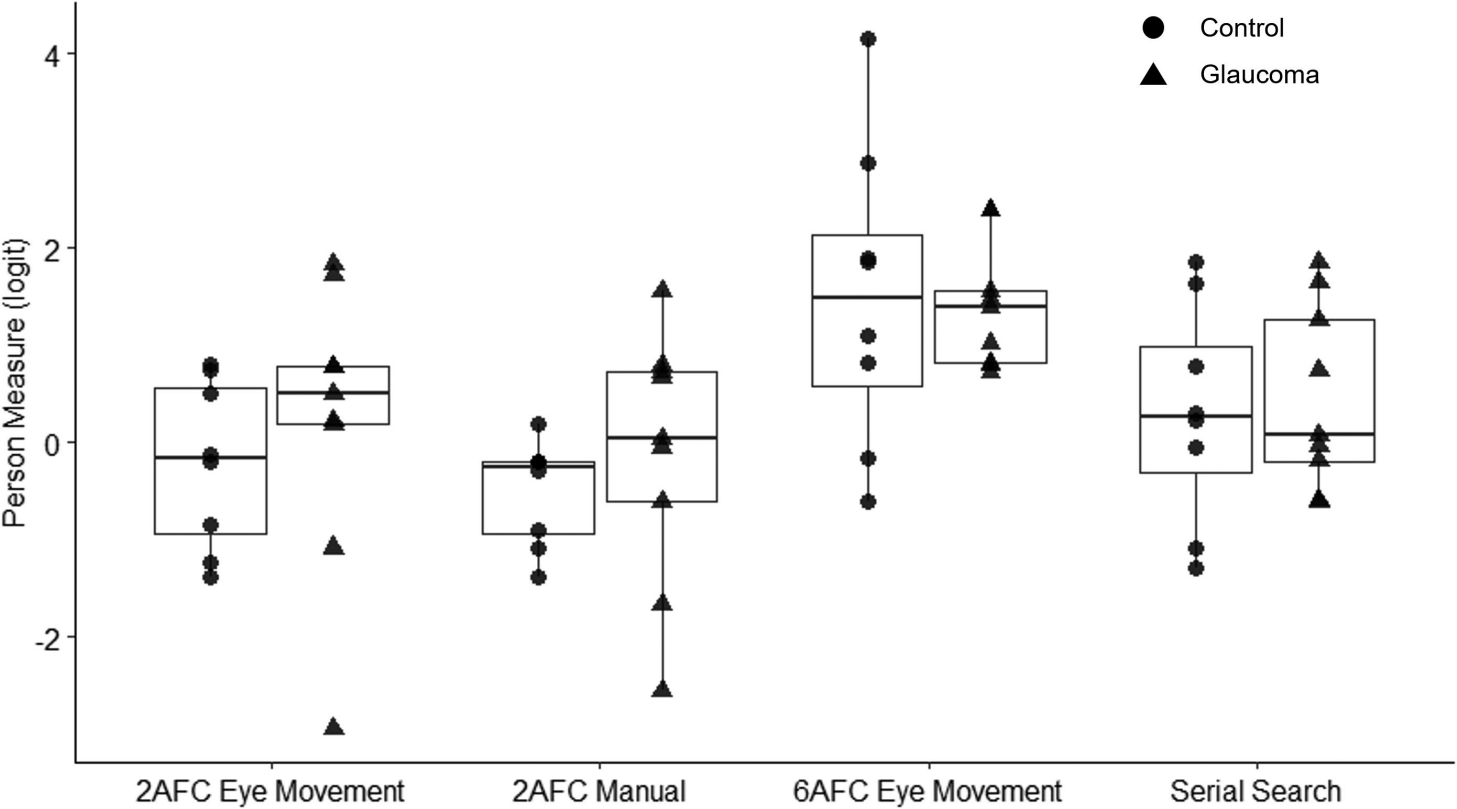
“Person measures” for 2AFC eye movement, 2AFC manual, 6AFC eye movement, and serial search paradigms. The *symbols* on the boxplots represent the individual “person measures” while the boxplots show the median and interquartile range. A higher score indicates a lower preference for the paradigm. Participants preferred the 6AFC eye movement paradigm the least out of all four paradigms.

Our final dependent variable was the person measure estimated through Rasch analysis. Figure 8 shows that both the control and glaucoma groups least favored the 6AFC eye movement paradigm but showed equal preference for the other three paradigms. Results revealed a significant main effect of paradigm *F*(3, 45) = 11.5300, *P* < .001), confirming differences in preference among the paradigms. There was neither a significant main effect of group (*F*(1, 45) = 0.35, *P* = 0.56) nor an interaction between paradigm type and group (*F*(3, 45) = 0.30, *P* = 0.83). The post hoc pairwise comparisons indicated significant differences between 2AFC and 6AFC eye movement (*t*(45) = −4.56, *P* < 0.01), 2AFC manual and 6AFC eye movement (*t*(45) = −5.56, *P* < 0.01), and 6AFC eye movement and serial search (*t*(45) = 3.37, *P* = 0.01) paradigms.

Our questionnaire also asked participants whether they had understood the instructions. This question was analyzed separately to assess whether the instructions for each test were sufficiently clear. The mean score ranged between 4.75 and 4.8 for all paradigms, with 5 indicating full comprehension (SD range is between 0.09 and 0.1). There was no difference between paradigms in terms of comprehension (all *P*s *>* 0.05).

## Discussion

Our main findings are that (1) participants with preperimetric glaucoma show elevated visual crowding compared to healthy age-matched controls, (2) a 2AFC manual response paradigm was most effective in revealing this elevation, (3) neither group was able to perform a 6AFC eye movement response paradigm, and (4) a serial search paradigm incorporating continuous eye movements was the fastest paradigm to assess thresholds in both control and glaucoma groups, yet showed the smallest crowding magnitude differences.

### Participants With Preperimetric Glaucoma Exhibit Elevated Visual Crowding

In this study, we demonstrate that crowding is elevated in participants with preperimetric glaucoma compared to healthy age-matched controls where the stimulus was presented in their periphery binocularly. This aligns with the conclusions of previous studies. Ogata et al.^9^ demonstrated that when measured monocularly, patients with glaucoma with early to mild visual field loss experienced heightened peripheral crowding compared to age-matched healthy controls even though the stimulus was exclusively presented in intact areas of the visual field. Shamsi et al.^10^ also previously demonstrated that parafoveal crowding was higher in the worse compared to the better eye of patients with glaucoma when tested monocularly. Moreover, according to their results, binocular crowding was also elevated in the parafovea of patients with glaucoma compared to age-matched controls. The fact that elevated crowding is prominently present during binocular viewing in patients with glaucoma suggests that this might affect both their daily visual function as well as the results of clinical assessment.

Our findings, which reveal elevated crowding even in the absence of a demonstrable visual field loss, lend further support to the suggestion by Ogata et al.^9^ that increased crowding in patients with glaucoma may be linked to early retinal ganglion cell loss. Ogata et al.^9^ found a correlation between decreased RNFL thickness and crowding extent in patients with glaucoma. In our study, we replicated these results by showing a correlation between RNFL thickness and crowding magnitude. It has been shown that in patients with glaucoma, lower RNFL thickness may be found in regions unaffected by apparent visual field loss.^18^ Moreover, previous literature shows a link between reduced ganglion cell density, indicated by a thinning of the RNFL and GCL, and an increased crowding zone.^11^^,^^17^ Additionally, previous literature shows increased enlarged areas of summation due to reduced ganglion cell density in glaucomatous vision.^16^ Therefore, the enlarged areas of summation in the periphery in glaucomatous vision^16^ might explain why we observe elevated crowding in patients with preperimetric glaucoma.

### Eye Movement Paradigms Prove Challenging in Elderly

Even though we show a general elevation of crowding in patients with glaucoma, the observed elevated crowding in the glaucoma group was only statistically significant in the 2AFC paradigm, where participants responded using the keyboard. In the serial search paradigm and the 2AFC paradigm with eye movement responses, the difference in crowding magnitude between glaucoma and control groups did not reach significance. This is likely caused by the number of participants who had difficulty performing the task in these paradigms. This is implied by more of them reaching floor orientation thresholds, leading to an inaccurate and probably underestimation of crowding magnitude. On top of not being able to differentiate the glaucoma and control groups in serial search and 2AFC eye movement paradigms, we also observed that both the glaucoma group and the control group were unable to perform the 6AFC paradigm with the eye movement responses. What may have caused the difficulty of the eye movement–based paradigms? Studies have shown that performing eye movements can be attentionally costly.^39^^,^^40^ Moreover, it is known that attention declines with age.^41^^–^^43^ Thus, issues in planning and executing the saccadic eye movements and attentional ones might have added too many non-crowding-related errors, causing an overall increase in orientation discrimination thresholds in the eye movement response paradigms.^44^ To probe into this possibility, we considered a well-performing participant in the 2AFC eye movement paradigm who had an actual threshold of 5.4 in the isolated condition and 22.8 in the flanked condition, resulting in a crowding magnitude of 4.2. To simulate the influence of random erroneous responses on the thresholds, we randomly replaced 20 correct responses with erroneous ones. We found that their threshold in the isolated condition increased to 31 and in the flanked condition to 42.3. Consequently, the crowding magnitude for this participant decreased to 1.4. This suggests that attentional and eye movement issues may indeed have affected a substantial number of participants.

Additionally, the need to distribute spatial attention in the serial search and 6AFC paradigms might have put an additional attentional strain on the participants, leading to floor performance in almost every participant. It has previously been shown that the more individuals need to distribute their spatial attention, the worse they become at perceptual tasks requiring attention,^45^^,^^46^ which is exacerbated with age.^42^^,^^43^ In the 2AFC paradigms, participants did not need to distribute their attention since covertly attending one location would be sufficient regardless of whether the target was presented in that location or the opposite one. However, in the 6AFC and serial search paradigms, covertly attending to one location would not guarantee detecting the location of the target.

Moreover, Coeckelbergh et al.^47^ reported that older adults display smaller attentional fields compared to younger adults, especially if the stimulus was presented briefly into the periphery. This may explain why more participants had difficulty performing the 6AFC paradigm compared to the serial search paradigm. Both paradigms required participants to distribute their spatial attention. However, in the 6AFC paradigm, the stimulus was presented only for a brief amount of time. In contrast, in the serial search paradigm, participants were allowed to explore their visual field as long as they required, without making eye movements. The short presentation of the stimulus, combined with participants’ decreased ability to distribute their attention, might both explain why we observed marked floor effects in the 6AFC paradigm and why participants least preferred the 6AFC paradigm.

In our previous study^28^ with younger participants, we found more variability and increased threshold within the eye movement paradigms, specifically in the 6AFC and the serial search paradigms. We argued that attentional processes may have increased the variability in these paradigms. In this study, we additionally show that these paradigms lead to higher thresholds in older adults, a population known to have decreased attentional abilities. Overall, this suggests that attentional processes likely affected the threshold measurements per se, rather than the crowding mechanism itself. Had attention directly influenced crowding, we would have expected reduced crowding in the 2AFC paradigms compared to the 6AFC and serial search paradigms. That is because crowding can be reduced when attention is directed to the target.^48^^–^^50^ As discussed above, participants could focus their attention on one location in the 2AFC paradigm. This was not possible in the 6AFC and serial search paradigms. However, note that we do not exclude that attention influences crowding in these paradigms for elderly or glaucomatous populations. The inaccurate assessment of crowding magnitude in the 6AFC and serial search paradigms does not allow drawing any firm conclusions on this matter.

In our previous study,^28^ we showed that all paradigms using eye movement responses resulted in faster assessment of crowding compared to the paradigms using manual responses. In this study, the response type did not affect the assessment time in the 2AFC paradigms. It has been shown that age significantly affects the peak velocity and mean velocity of saccades, and older people have lower peak and mean velocity.^51^ Slower saccades and decreased attention in older adults might be why we do not see a faster assessment time in the 2AFC eye movement response paradigm compared to the 2AFC manual response paradigm. The rapidity of the serial search paradigm, on the other hand, can be explained by the nature of this paradigm, where participants did not have to return to the fixation point after every trial. Instead, they could make continuous saccades from the location of one target to the next. Therefore, the time advantage of a continuous response measure can still be observed in measuring thresholds.

In conclusion, even though eye movements as a response measure are useful for rapidly assessing crowding in a healthy young population,^27^^,^^28^ their use in elderly participants may complicate rather than facilitate crowding assessments.

### Limitations and Future Directions

As discussed above, paradigms utilizing eye movements resulted in increased thresholds with a considerable number of participants even reaching floor-level threshold values in both isolated and flanked conditions. This prohibited these paradigms from distinguishing the preperimetric glaucoma group from the controls on the basis of crowding magnitude. As we argued, the limitation may have come from attentional issues adding too many erroneous responses, potentially stemming from the inherent difficulty of these paradigms. Possibly, adjusting the difficulty of the baseline (isolated) condition of a paradigm may ameliorate some of these. This might be done by manipulating the stimulus properties according to each individual's threshold performance to achieve a minimal baseline performance. As a consequence, more accurate estimates of crowding magnitudes might be obtained for all paradigms.^27^

Additionally, the specific contrasts and spatial frequency used in this study, as well as the fixed eccentricity of 6°, introduce certain limitations. These fixed parameters may limit the generalizability of the findings, as varying the contrast, spatial frequency, or eccentricity could potentially alter the observed patterns of results. Future research should explore the effects of different contrast levels, spatial frequencies, and eccentricities to provide a more comprehensive understanding of the relationship between visual crowding and glaucoma.

As noted above, we could not clearly establish whether differences in attentional demand in the paradigms would have an effect on crowding magnitude in older adults or glaucomatous populations, due to inaccurate assessment of crowding magnitude in the 6AFC and serial search paradigms. Suppose the baseline performance can be adjusted to reach a reliable individual assessment of crowding magnitude in these paradigms. In that case, the question of whether crowding magnitude is affected by attention can be reinvestigated, which is relevant since the attentional capabilities of participants can vary greatly among older adults.^52^

## Conclusions

In conclusion, our study reveals that patients with preperimetric glaucoma exhibit elevated crowding. Importantly, the effectiveness of different paradigms in assessing crowding varied, with the 2AFC manual response paradigm proving most effective in capturing the observed elevation. However, challenges arose with eye movement–based paradigms, potentially due to age-related declines in attentional and saccadic capabilities. It is important to note that while these challenges present complexities in task performance, they do not negate the utility of eye movement–based assessments entirely. Rather, they underscore the need for further refinement and consideration of individual capabilities when employing such paradigms. In summary, our findings underscore both the value and the complexity of the efficient evaluation of peripheral crowding in patients with preperimetric glaucoma.

## Supplementary Material

Supplement 1
