# Supplementary material for: Assessing Visual Crowding in Participants With Preperimetric Glaucoma Using Eye Movement and Manual Response Paradigms
Source: Transl Vis Sci Technol. 2024 Sep 5;13(9):8. doi: 10.1167/tvst.13.9.8 (PMC11379081; doi:10.1167/tvst.13.9.8)
Supplement: Supplement 1 [file tvst-13-9-8_s001.pdf]

Table S1. The paradigm questionnaire to asses preference. Please note that questions were originally asked in German.

| | 2AFC Eyes | 2AFC Manual | 6AFC Eyes | Serial Search |
|---|---|---|---|---|
| How would you assess the difficulty of the paradigm?<br>1. not difficult<br>5. very difficult | | | | |
| How tiring was the paradigm?<br>1. not tiring<br>5. very tiring | | | | |
| How demanding was the paradigm?<br>1. not demanding<br>5. very demanding | | | | |
| How much attention did the paradigm require?<br>1. Not much<br>5. Very much | | | | |
| How much effort did the paradigm require<br>1. Not much<br>5. Very much | | | | |
| Which task do you prefer the most, rank them from 1 to 4? 1 being the most preferred task, and 4 being the least preferred task. | | | | |
| How well did you understand the instructions?<br>1. Not well<br>5. Very well | | | | |

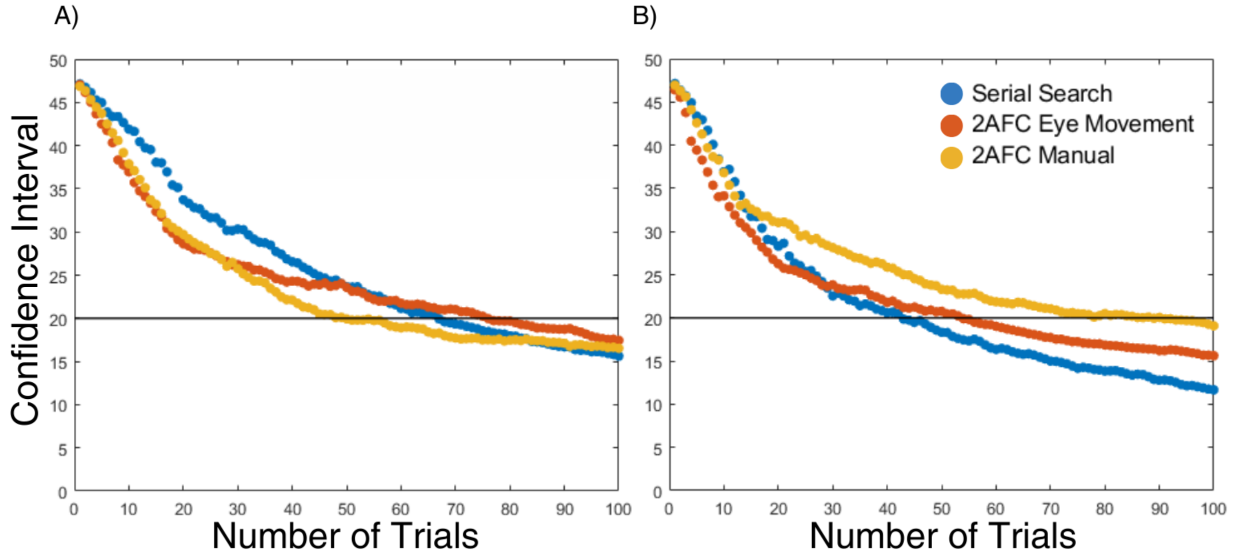


Figure S1. Change in confidence interval for the thresholds after each trial for each paradigm and condition. (A): isolated condition (B): flanked condition. In order to appropriately consider potential variations in the effectiveness of the paradigms, we derived a posteriori cut-off confidence interval (CI) by inspecting the mean CI plots of all paradigms. Based on this, a cut-off CI value of 20 was chosen. The black lines represent the selected CI cut-off.

Table S2. *F and p values of main and interaction effects of the ANOVA's on thresholds and crowding magnitude for comparison of re-estimated and original values.*

| **Effects** | **Threshold** | | **Crowding Magnitude** | |
|---|---|---|---|---|
| | ***F*** | ***p*** | ***F*** | ***p*** |
| Threshold Calculation | 3.69 | 0.07 | 2.42 | 0.14 |
| Threshold Calculation*Group | 0.64 | 0.43 | 4.36 | 0.051 |
| Threshold Calculation*Condition | 0.06 | 0.81 | NA | NA |
| Threshold Calculation*Condition*Group | 2.49 | 0.13 | NA | NA |
| Threshold Calculation*Paradigm | 10.24 | 0.001 | 0.34 | 0.72 |
| Threshold Calculation*Paradigm*Group | 0.72 | 0.46 | 1.54 | 0.23 |
| Threshold Calculation*Paradigm*Condition | 4.91 | 0.02 | NA | NA |
| Threshold Calculation*Paradigm*Condition*Group | 1.92 | 0.17 | NA | NA |

Following the adjustment of the number of trials based on this CI, we proceeded to verify whether such adjustments would impact the thresholds and crowding magnitude. This was achieved by comparing the original threshold and crowding magnitude values to the recalculated ones. Both ANOVAs yielded results indicating that the re-estimated values were not significantly different from the original values.

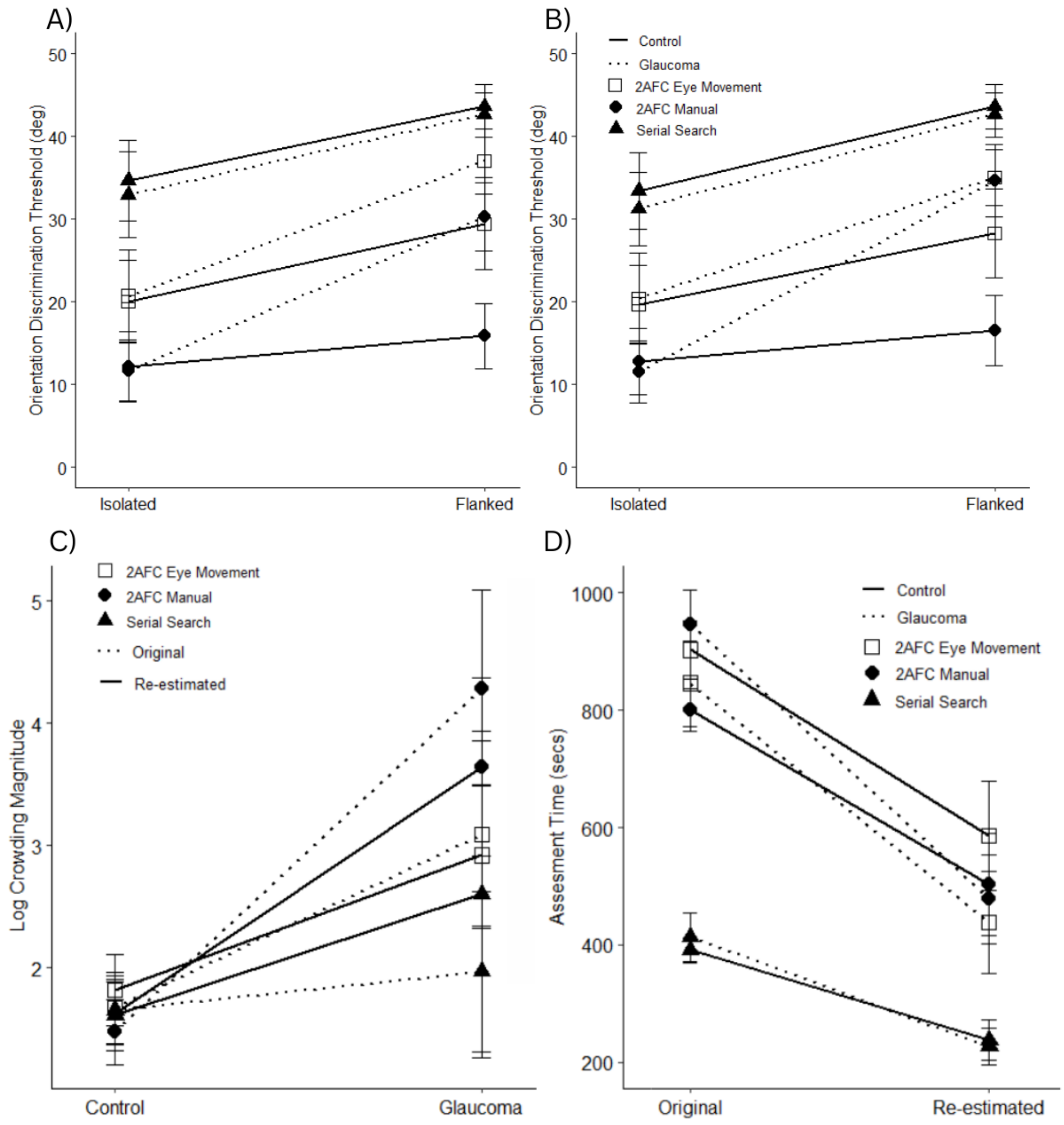


Figure S2. Comparison of original and re-estimated values. (A) shows the original orientation thresholds for all three paradigms while (B) shows the re-estimated thresholds. (C) shows the log crowding magnitudes calculated using original (dotted lines) and re-estimated (solid lines) values.

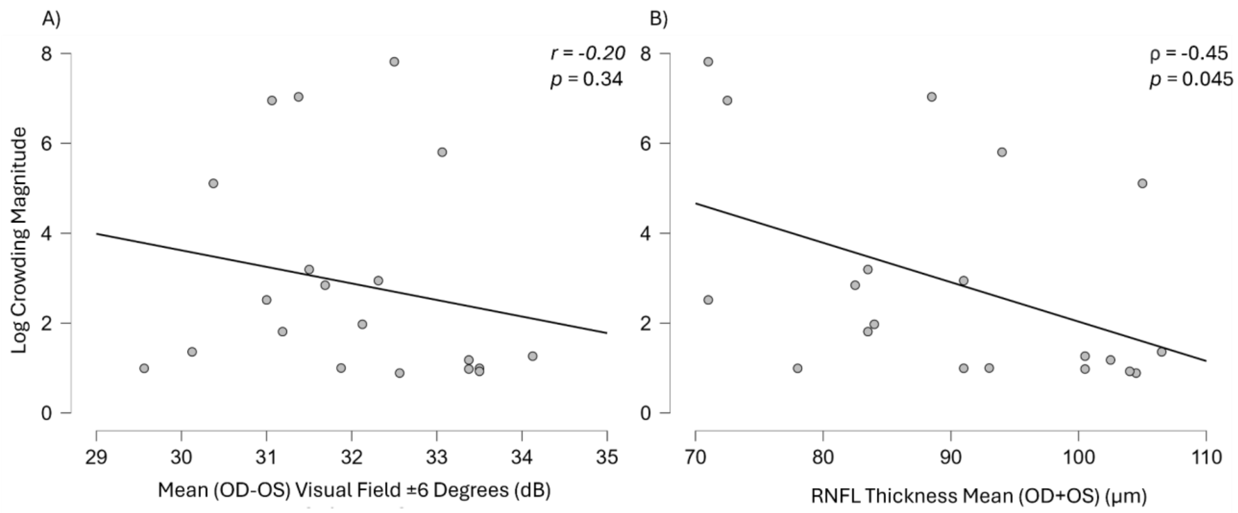


Figure S3. Correlation between crowding magnitudes in the 2AFC manual paradigm and localized sensitivities (A) as well as RNFL thickness (B).

A) The localized sensitivities were calculated using the ±3-degree and ±9-degree local sensitivity values of both the left and right eyes to achieve sensitivities for (±6,0). These values were obtained using HFA 24-2 as described in the methods section. Because the stimulus was presented binocularly at both -6 and +6 degrees horizontally, the average of ±6 degrees, as well as both eyes, were used to perform the correlation analysis. After the Shapiro-Wilk test for bivariate normality, Pearson correlation was performed We found no significant correlation between the localized sensitivities and crowding magnitude.

B) The RNFL thickness values were calculated by averaging the RNFL thickness values of both eyes. Note that the OCT scan of the patients was not done on the day of the experiment; the RNFL thickness values were obtained on dates ranging from a year before to a year after the experiment. Therefore, the results of this analysis should be approached with that in mind. Before the analysis, the Shapiro-Wilk test for bivariate normality was conducted. Due to the non-normal nature of the data, a Spearman correlation was conducted. We found a significant negative correlation between the crowding magnitude and mean RNFL thickness.
